# The relationship between postoperative parathormone suppression and surgical cure in primary hyperparathyroidism

**DOI:** 10.3389/fendo.2025.1629719

**Published:** 2025-07-30

**Authors:** Mehmet Taner Ünlü, Nurcihan Aygun, Mehmet Kostek, Ozan Caliskan, Mehmet Uludag

**Affiliations:** ^1^ Department of General Surgery, Şişli Hamidiye Etfal Training and Research Hospital, Istanbul, Türkiye; ^2^ Department of General Surgery, Ümraniye Training and Research Hospital, Istanbul, Türkiye

**Keywords:** primary hyperparathyroidism (pHPT), persistent disease, parathyroidectomy, parathormone suppression, parathyroid adenoma, surgical cure

## Abstract

**Introduction:**

In primary hyperparathyroidism(pHPT), suppression in other glands due to autonomy of pathological gland is frequently observed. In this retrospective study, we aimed to evaluate contribution of suppression of remaining parathyroid glands in pHPT in predicting surgical cure.

**Methods:**

We retrospectively analyzed data from patients diagnosed with pHPT and operated at our institution between 2014 and 2022. Patients who demonstrated either a decrease of more than 50% in intraoperative parathormone levels or a normal parathormone (PTH) level at the 6th postoperative hour were included. Patients were categorized into two groups based on their PTH levels at the 6th postoperative hour: those with PTH suppression (PTH < 15 ng/L) and those without (PTH > 15 ng/L). We analyzed the outcomes in terms of persistent disease and biochemical markers.

**Results:**

Among 196 patients who met the inclusion criteria, 124 exhibited PTH suppression while 72 did not. Persistent disease was significantly more common in the non-suppressed group (19.4% *vs*. 5.65%, p<0.001). Furthermore, postoperative PTH suppression strongly correlated with surgical cure, indicated by a significant difference in the rate of normocalcemia after 6 months. Excised parathyroid tissue volumes were determined significantly lower in group 1 compared to group (0.85 ± 0.88cm3*vs*2.04 ± 3.79cm3,p=0.035, respectively).There was no significant difference between two groups in terms of gender, preoperative Ca, magnesium, vitamin D and postoperative Ca levels.

**Conclusion:**

Early postoperative PTH suppression is highly associated with surgical cure. The rate of pPHPT is significantly higher in non-suppressed patients. Therefore, in follow-up strategies of postoperative patients, the possibility of a remnant pathological gland should be considered especially in those without early PTH suppression.

## Introduction

Primary hyperparathyroidism(pHPT) is one of the most common endocrine pathologies and the most common cause of hypercalcemia. It’s only curative treatment is surgery (1). Although surgical success rate is over 95% in experienced centers, surgical failure is still the most common complication (1, 2). The most common causes of surgical failure at initial exploration are multiglandular disease such as double adenoma or four-gland hyperplasia and ectopic glands (3) In the literature, rate of persistent disease after parathyroidectomy is reported to be between 2% and 22%, while rate of recurrent disease between 1% and 15% (2, 4).

Parathyroid hormone (PTH) has a critical role in regulation of calcium (Ca) homeostasis. PTH secretion from parathyroid glands is regulated by negative feedback due to small changes in circulating ionized Ca via Ca-sensitive receptors(CaSR) on surface of parathyroid glands. While decrease in serum Ca level stimulates PTH secretion with CaSR inactivation, increase in serum Ca level inhibits PTH secretion with CaSR stimulation (5). In pHPT, it has been demonstrated that there is a deterioration in set-point value of Ca concentration inhibiting PTH release or a deterioration in Ca suppression mechanism over parathyroid glands (6). This mechanism is impaired in all pathological parathyroid glands (7). In pathological glands, suppressive effect of Ca on PTH secretion is impaired but not completely eliminated. Sensitivity of parathyroid cells to Ca and suppressive effect of Ca on PTH secretion decrease (8).

Rate of PTH suppression in response to oral Ca challenge is lower in pHPT patients compared to normal population. In normal individuals, PTH secretion is suppressed to almost undetectable levels within first hour of oral Ca intake (9). In pHPT patients, it has been demonstrated that decreased suppressibility of PTH in response to Ca in preoperative period gets normalized on first postoperative day and remaining parathyroid glands respond normally to changes in extracellular Ca (6).

For the past three decades, intraoperative parathyroid hormone(ioPTH) assay during pHPT surgery has been based on the assumption that PTH is rapidly eliminated from plasma due to its short half-life and remaining normal glands are functionally suppressed (10). The use of ioPTH measurement is increasingly being employed to predict surgical cure and reduce operative failures in parathyroidectomy for pHPT. While ioPTH measurement is effective in reducing persistent disease, surgical failure remains a major challenging problem (11). Studies assessing the false-positive and false-negative rates of ioPTH method continue to appear in the literature. In a meta-analysis conducted by Medas et al., encompassing 18 studies on the topic, the concordance rate for the ioPTH method was reported to range from 81.3% to 100% (11). In patients where at least one pathological gland has been removed, the most common cause of persistent disease is the presence of a remnant pathological gland due to multiglandular disease.

While ioPTH measurement provides excellent predictions of surgical success in single adenoma cases, rates of false positives are high in multiglandular diseases and in cases with double adenomas (22%, 47%,respectively). Particularly in multiglandular diseases, even if ioPTH falls sufficiently and below normal range’s lower limit, it does not guarantee that all pathological parathyroid glands have been completely removed (12). This situation indicates that additional data are needed to predict surgical cure. Although current guidelines recommend a minimum follow-up period of six months after parathyroidectomy to evaluate surgical outcomes and potential complications, there are no clear recommendations regarding the optimal frequency of follow-up visits (1).

Similarly, among our patients who underwent unilateral neck exploration (UNE) or minimally invasive parathyroidectomy (MIP) and exhibited a sufficient ioPTH decline—defined as a decrease of more than 50% from the highest pre-excisional PTH level during ioPTH monitoring—persistent disease was detected in some cases during postoperative follow-up. We observed that PTH values at postoperative 6th hour were in normal range in some of them while in others it was under lower limit of normal reference range. In particular, it suggests that patients with suppressed PTH are more likely to have normal parathyroid glands. Main cause of persistent disease is the presence of a pathological remnant gland. PTH may not be suppressed in presence of a possible pathological remnant gland.

Present study was planned with hypothesis that rate of persistent disease may be lower in those with early postoperative PTH suppression and PTH suppression may predict surgical cure. The association between PTH suppression in the early postoperative period and the subsequent development of persistent disease was evaluated, and its potential contribution to determining the optimal follow-up schedule for patients was assessed.

## Material and methods

Data of patients with pHPT who were operated at our clinic between 2014 and 2022 were retrospectively evaluated. Approval for data evaluation was obtained from our hospital’s local ethics committee, and data were used in accordance with the Declaration of Helsinki. Informed consent for use of their data in scientific studies was obtained from all patients preoperatively.

Inclusion criteria: Patients with one or two positive imaging methods who underwent focused surgery or unilateral exploration were included. For patients with a single positive or two positive imaging result, the operation was terminated if ioPTH levels showed a 50% drop from the pre-excision value at 10 or 20 minutes post-excision (Modified Miami criterion) (13). At least 2 parathyroid glands on the contralateral neck side were not explored to protect them from the potential traumatic effects of surgical manipulation. A successful surgical outcome was defined as a reduction in ioPTH levels by more than 50%, or as a PTH level measured at the 6th postoperative hour falling below the upper limit of the laboratory reference range. Patients who demonstrated either a decrease of more than 50% in ioPTH levels or a normal PTH level at the 6th postoperative hour were included.

Exclusion criteria: Patients with pHPT who underwent bilateral neck exploration with two negative or discordant imaging results, those with familial primary hyperparathyroidism or multiple endocrine neoplasia or normocalcemic pHPT, those converted from focused surgery to bilateral exploration, patients who had previously undergone thyroid or parathyroid surgery, and those who did not consent to the use of their data in scientific studies were excluded. Patients who did not exhibit a sufficient decline in ioPTH levels, those who did not undergo ioPTH monitoring, and individuals with incomplete data were excluded from the study.

The diagnosis of pHPT was biochemically confirmed in all patients. All patients underwent routine preoperative sestamibi parathyroid scintigraphy and ultrasonography(USG). All USGs were performed by an experienced radiologist. In early postoperative period, before discharge, patients exhibited normocalcemia and/or normoparathyroidism, hypoparathyroidism(PTH<65ng/mL).

Retrospectively, preoperative levels of Ca, phosphorus, magnesium, PTH, 25(OH)vitaminD3 were evaluated. Intraoperative pre-excision PTH levels, post-excision 10 and 20-minute PTH levels, and postoperative 6th hour PTH, Ca, levels were also assessed, as well as postoperative day 1 Ca level. Additionally, excised parathyroid volumes were evaluated. Volumes of the adenomas were calculated according to the diameters obtained from pathology reports with the formula V(Volume)(mm3)=L(Length)(mm)×W(Wide)(mm)×H(Height)(mm)×π/6 (14).

Based on PTH values at postoperative 6th hour, patients were divided into two groups: Group 1, patients with PTH suppression(PTH<15 ng/L); Group 2 without PTH suppression(PTH>15 ng/L). Groups were compared regarding preoperative and early postoperative Ca values, imaging characteristics, pPHPT development.

Achieving normocalcemia for at least 6 months was defined as a surgical cure. Persistent disease was defined as recurrent hypercalcemia within 6 months postoperatively, recurrent disease was defined as recurrence of hypercalcemia after at least 6 months of normocalcemia (1).

### Statistical analysis

SPSS Statistics Version 25 program was used for statistical analyses. Descriptive statistical methods(mean, standard deviation, median, first quartile, third quartile, frequency, percentage, minimum, maximum) were used to evaluate study data. “Mann-Whitney U” test was used for comparisons between two groups of quantitative variables that did not exhibit normal distribution. “Pearson chi-square test” and “Fisher’s Exact Test” were used for comparisons of qualitative data. Spearman correlation analysis was utilized to assess relationships between quantitative variables. Diagnostic screening tests(sensitivity, specificity, positive predictive value, negative predictive value, accuracy) and ROC analysis were used to determine predictive value for parameters.

## Results

Among patients operated on due to pHPT, 196 patients who met inclusion criteria were evaluated. While postoperative PTH suppression (group 1) was detected in 124 of 196 patients, no PTH suppression (group 2) was detected in 72 patients. In group 2 compared to group 1; mean age (56 ± 14 *vs* 51.9 ± 11.8,respectively) was significantly higher in group 2 (p=0.037). In group 1 compared to group 2, preoperative PTH value (144.96 + 121.47ng/L *vs* 268.96 + 239.80ng/L,p=0.00, respectively), were significantly lower (**Table 1**). Excised parathyroid tissue volumes were determined significantly lower in group 1 compared to group 2 (0.83 ± 0.85cm3 *vs* 2.21 ± 3.92cm3,p=0.0044, respectively). Persistent disease was detected in 21 (10.7%) of 196 patients in study. Persistent pHPT was significantly lower in group 1 than group 2 [5.65% *vs* 19.4%, p<0.001; OR:0.248 (95% CI:0.095-0.648)] (**Table 1**).

**Table 1:** Demographic, preoperative and postoperative features of patients.

		All Patients (n=196)	Group 1 (n=124)	Group 2 (n=72)	p-value
Gender n (%)	M	29 (14.8%)	19 (15.3%)	10 (13.9%)	0.949
	F	167 (85.2%)	105 (84.7%)	62 (86.1%)	
Age	year	53.37 ± 12.78 (16-83)	51.85 ± 11.82 (24-83)	55.99 ± 13.97 (16-83)	0.037
Preop Ca Mean±SD Min - Max	(8.8 – 10.6 mg/dL)	11.07 ± 0.77 (8.87-13.71)	11.02 ± 0.65 (9.52-12.97)	11.17 ± 0.94 (8.87-13.71)	0.219
Preop Mg Mean±SD Min - Max	(1.8 – 2.6 mg/dL)	1.97 ± 0.23 (1.04-2.51)	2.00 ± 0.20 (1.31-2.51)	1.93 ± 0.28 (1.04-2.44)	0.096
Preop P Mean±SD Min - Max	(2.5 – 4.5 mg/dL)	2.66 ± 0.54 (1.10-4.20)	2.69 ± 0.58 (1.10-4.20)	2.62 ± 0.46 (1.76-4.00)	0.386
Preop ALP Mean±SD Min - Max	(43–115 U/L)	102.88 ± 48.18 (37.0-313.0)	101.74 ± 42.23 (37.0-204.0)	105.03 ± 58.60 (45.0-313.0)	0.790
Preop PTH Mean±SD Min - Max	(15–65 ng/L)	190.82 ± 184.25 (51.6-1337.1)	144.96 ± 121.47 (51.6-1302.0)	268.96 ± 239.80 (60.2-1337.1)	0.000
Preop Vitamin D Mean±SD Min - Max	(20 – 100 µg/L)	21.78 ± 51.91 (3.0-657.0)	25.60 ± 63.94 (3.2-657.0)	14.70 ± 7.72 (3.0-35.4)	0.092
Persistence / Cure	Persistence	21 (10.7%)	7 (5.6%)	14 (19.4%)	0.0056
	Cure	175 (89.3%)	117 (94.4%)	58 (80.6%)	
Postop 6^th^ hour PTH Mean±SD Min - Max	(15–65 ng/L)	16.76 ± 13.60 (3.3-75.6)	9.43 ± 3.07 (3.3-14.8)	29.39 ± 15.35 (7.68-75.6)	0.000
Postop 6^th^ hour Ca Mean+SD Min - Max	(8.8 – 10.6 mg/dL)	9.72 ± 0.91 (7.39-12.39)	9.70 ± 0.85 (7.8-11.6)	9.74 ± 1.01 (7.39-12.39)	0.755
Postop 1^st^ day Ca Mean±SD Min - Max	(8.8 – 10.6 mg/dL)	8.88 ± 0.66 (6.84-10.57)	8.84 ± 0.66 (7.3-10.57)	8.97 ± 0.66 (6.84-10.5)	0.189
Excised Parathyroid Tissue Volume Mean±SD Min - Max	(cm3)	1.34 ± 2.55 (0.03-25.91)	0.83 ± 0.85 (0.03-4.40)	2.21 ± 3.92 (0.03-25.90)	0.0044
Ultrasonography	Positive	181 (92.3%)	112 (90.3%)	69 (95.8%)	0.263
	Negative	15 (7.7%)	12 (9.7%)	3 (4.2%)	
Scintigraphy	Positive	173 (88.3%)	107 (86.3%)	66 (91.7%)	0.370
	Negative	23 (11.7%)	17 (13.7%)	6 (8.3%)	
Single Positive Imaging	(n)	32	23	9	No p Value
Double Positive Imaging	(n)	158	95	63	
Discordant Imaging Methods	(n)	3	3	0	

n, number; M, Male; F, Female; Ca, Calcium; SD, Standard Deviation; min, minimum; max, maximum; Mg, Magnesium; P, Phosphorus; ALP, Alkaline Phosphatase; PTH, Parathyroid hormone.

While PTH suppression was detected in 117 (66.95%) of 175 surgically cured patients, no suppression was observed in 58 (33.1%) patients. There was no significant difference between the two groups in terms of gender, preoperative Ca, magnesium, vitamin D, postoperative Ca levels (**Table 1**).

**Figure 1:**
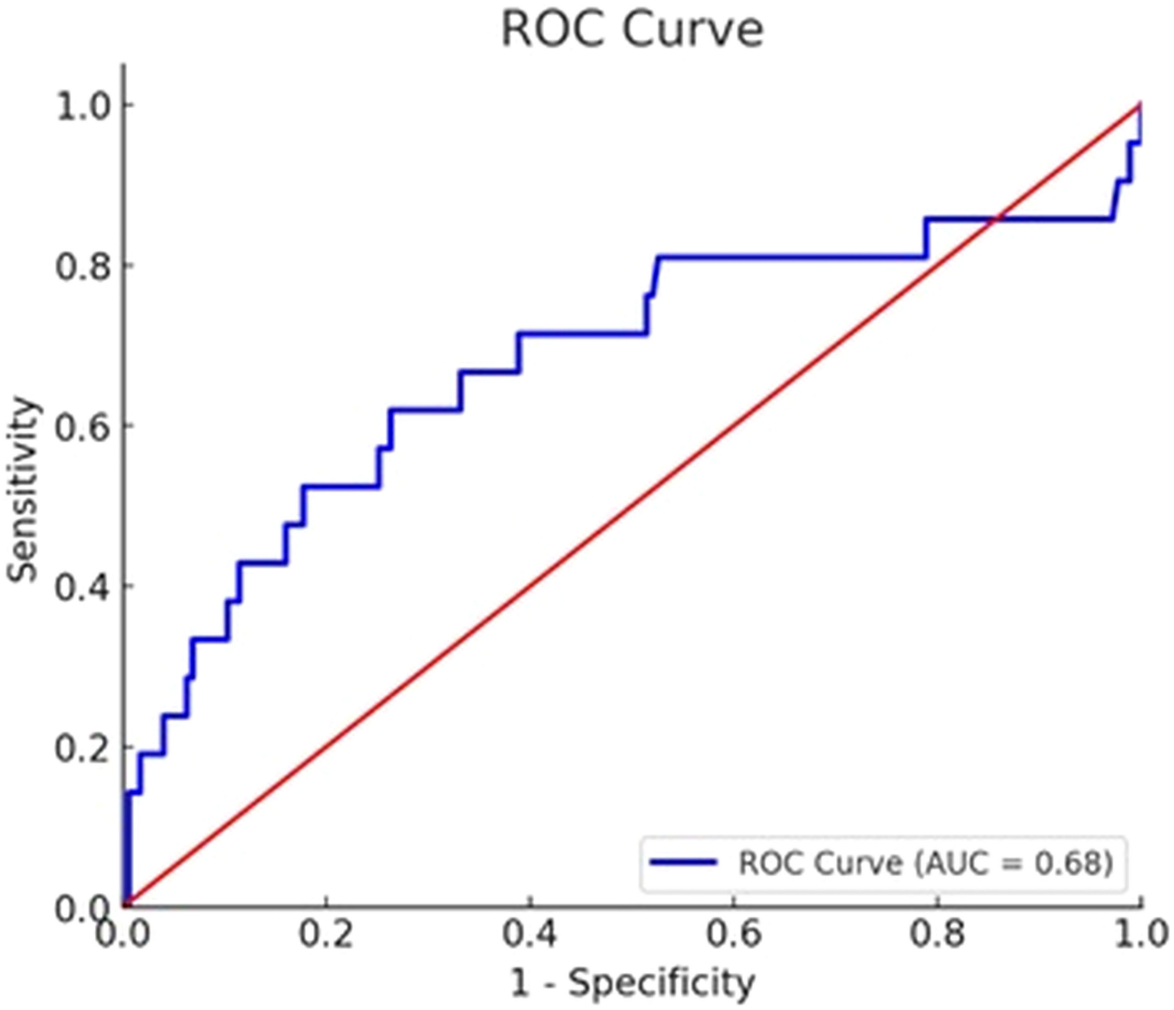
ROC Curve (ROC Curve for 6th Hour PTH in Predicting Surgical Cure. AUC = 0.68 (95% CI: 0.545–0.809), p = 0.0059.).

According to our results, postoperative 6th hour PTH value less than 18 ng/L predicts surgical cure with 61.9% sensitivity and 73.7% specificity (**Table 2**, **Figure 1**).

**Table 2:** ROC analysis for postoperative 6th hour PTH in predicting surgical cure PTH: parathyroid hormone.

Cut-off Value (ng/L)	Sensitivity (%)	Specificity (%)	AUC (95% CI)	p-value
18	61.9	73.7	0.68 (0.545–0.809)	0.0059

ROC, Receiver Operating Characteristic; AUC, Area Under the Curve.

The optimal cut-off value was determined as 18 ng/L, with a 95% confidence interval of 11.6–33.9 ng/L, using ROC analysis. The area under the curve (AUC) was calculated as 0.68 (95% CI: 0.545–0.809), indicating a fair discriminatory ability, and this result was statistically significant (p = 0.0059) based on comparison of mean PTH levels between persistent and cured patients using the independent samples t-test (**Table 2**, **Figure 1**).

In addition to ROC analysis, the clinical validity of this cut-off was assessed using Fisher’s Exact Test, revealing a statistically significant association between the cut-off-based classification and actual surgical outcome (p = 0.0017). The odds ratio was calculated as 4.56 (95% CI: 1.78–11.70), suggesting that patients with a postoperative PTH below 18 ng/L were over four times more likely to be cured compared to those with higher values (**Table 3**).

**Table 3:** Diagnostic Performance of Postoperative 6th Hour PTH Cut-off Value PTH: Parathyroid Hormone. .

Cut-off Value (ng/L)	PPV (%)	NPV (%)	Accuracy (%)	p-value (Fisher's Exact Test)
18	22.0	94.2	72.5	0.0017

PPV, Positive Predictive Value; NPV, Negative Predictive Value.

In the analysis of postoperative 6th hour PTH values, it was observed that among the patients with a PTH value less than 18 ng/L, a significant majority (129 out of 138) indeed experienced surgical cure, which is reflected in a high Negative Predictive Value (NPV) of 94.2%. This suggests that a low PTH value shortly after surgery is a good indicator of surgical success (**Table 3**).

Conversely, for those with a PTH value of 18 ng/L or higher, only 13 out of 59 patients did not achieve a surgical cure, resulting in a low Positive Predictive Value (PPV) of 22.0%. This indicates that higher PTH values post-surgery are less reliable in predicting the lack of surgical cure (**Table 3**).

The overall accuracy of using the 6th hour PTH value to predict surgical outcomes was found to be 72.5%, indicating a reasonable level of reliability for this test in a clinical setting. The results demonstrate the test’s strength in confirming surgical success but also highlight its limitations in ruling out unsuccessful surgeries based on higher PTH levels (**Table 3**).

## Discussion

In pathological parathyroid glands, the regulatory mechanism by which calcium suppresses PTH secretion is disrupted. PTH suppression response to Ca intake is significantly lower in pHPT patients compared to normal population (15). The difference in Ca suppression response between normal and pathological parathyroid glands has been suggested as a method to distinguish between normal individuals and patients with mild pHPT whose diagnosis is unclear. Both oral and intravenous Ca loading result in significantly higher PTH suppression in normal individuals compared to pHPT patients. In normal individuals, PTH secretion decreases below the reference range with Ca loading and may even be suppressed to undetectable levels (9, 16).

Depending on this physiopathological mechanism, we aimed to evaluate whether early PTH suppression contributes to predicting surgical cure. The rate of persistent disease in patients who underwent focused surgery or unilateral exploration was 10.7%. Among patients with early postoperative PTH suppression, persistence rate was 5.65%, significantly lower than 19.4% observed in patients without suppression. The risk of persistence was approximately 3.3 times higher in patients without suppression (p = 0.0149; OR: 3.28; 95% CI: 1.29–8.35).

In pHPT, a disruption in the set point value at which extracellular Ca ion levels inhibit PTH secretion, or a malfunction in the suppression mechanism of Ca on the parathyroid glands, is a significant part of the disease’s pathophysiology (6). In present study, surgical cure rate was 94.35% in patients with PTH suppression at the postoperative 6th hour, approaching the optimal goal of 95% cure rate. This is consistent with the suppression of normal parathyroid glands by high Ca levels.

In patients with parathyroid adenomas, dispersed cells prepared from *in vitro* pathological glands and normal parathyroid glands were incubated with various concentrations of Ca, and PTH secretion was evaluated. The set point for PTH secretion (defined as the concentration of Ca causing half of the maximal inhibition of PTH release) in cells taken from normal glands was found to be lower than in cells taken from adenomas. Additionally, in normal gland cells of patients with adenomas, both the set point and maximum PTH secretion at low Ca concentrations were found to be significantly lower compared to the normal glands of patients with normal Ca homeostasis (glands sampled during thyroidectomy) (17). Bergenfelz et al. found that although the effect of Ca on PTH suppression decreases in pathological glands in pHPT, after pathological gland is removed, normal parathyroid glands can respond normally to extracellular Ca changes by the first postoperative day (6). Despite rapid decline in hormone levels due to half-life of PTH after removal of pathological gland, Ca levels decrease within 1–2 days (12). We hypothesize that the time for resolution of postoperative PTH suppression may vary depending on patient’s level of hypercalcemia, set point level of normal glands, and whether postoperative Ca levels fall below this set point level.

In present study, persistent disease secondary to a remnant pathological gland was found in 5.65% of patients in suppression group. Despite reduced suppressibility of PTH secretion in pathological glands compared to normal glands, it has been previously shown that PTH secretion can be suppressed down to zero (8). In the same patient, multiple pathological glands may exhibit different growth patterns and secretory functions. As a result, a dominant pathological gland identified through imaging can mask the degree of hypercalcemia caused by another pathological gland with lesser hypersecretory function. Although the true frequency of this phenomenon is unknown, it has been reported in the literature in a few case reports as a rare entity (12, 18). In these studies, after removal of pathological gland or glands, persistent disease can occur when a suppressed pathological gland with a different set point level starts to enter a hypersecretory phase, even though ioPTH levels are adequate and below the reference lower limit (18). These suppressed pathological parathyroid glands were defined as sleeping parathyroid glands by Yavuz et al. (12). The pathophysiology of persistent disease following a transient ioPTH decline has not been definitively determined, and several alternative hypotheses have been proposed. These include the presence of a second pathological gland that gains activation after the excision of dominant tumor, iatrogenic damage to another pathological gland during the excision of first gland that later reactivates upon healing, and the hypothesis that peroperative hypersecretion of PTH due to surgical manipulation results in a decline in PTH levels that is deemed adequate relative to this newly elevated value (12). In order to distinguish these suppressed pathological parathyroid glands from suppressed normal glands, other methods are needed in addition to evaluation of PTH suppression. In their study, Siperstein et al. routinely performed bilateral neck exploration and found that if bilateral exploration was not performed in patients whose USG and scintigraphy results were consistent and who had adequate ioPTH reduction after focal exploration and removal of index gland, 16% of occult multiglandular disease could be missed (19).

Despite a decrease in ioPTH by 50% or more, the group without PTH suppression had a significantly higher rate of persistent disease, which was found to be 19.4% in group 2 in present study. We advocate that in these patients, inadequate PTH suppression due to remaining pathological glands after removal of the preoperatively determined pathological gland may be reason for persistence. Previous studies have shown that Ca suppression tests have revealed significantly lower PTH suppression in patients with pHPT compared to healthy individuals (8, 9, 15, 16). Titon et al. reported that the intravenous standardized short-term PTH suppression test appears reliable in distinguishing healthy individuals from patients with pHPT. They found that PTH levels of 14 ng/mL at the end of the loading phase (<14ng/mL in healthy individuals, >14ng/mL in pHPT patients) and 23 ng/mL three hours later (<23ng/mL in healthy individuals, >23ng/mL in pHPT patients) were indicative (16). Heath et al. evaluated occult parathyroid hyperplasia in patients with MEN2a who were normocalcemic and had normal PTH levels, but were found to have parathyroid hyperplasia during surgery. They assessed suppression with Ca infusion test and found significantly lower PTH suppression in MEN2a patients compared to normal individuals and MEN2b individuals. Their findings indicated that suppression was impaired even in occult hyperplasia cases (20). These findings are consistent with data of persistent patients in the second group of the study, supporting that PTH suppression can often be impaired even with occult secondary remnant pathological glands.

Despite the belief that PTH secretion in normal glands is suppressed in patients with pHPT, some researchers have demonstrated that secretion function of normal parathyroid glands may not necessarily be completely suppressed even in the presence of pHPT (21, 22). Additionally, it has been suggested that ioPTH monitoring indicates that normal parathyroid glands are not completely suppressed (23). Bergenfelz et al. found that in patients with pHPT, normal parathyroid glands continued to secrete PTH within the first 60 minutes after adenoma removal, despite no hypocalcemic stimulus and exposure to prolonged hypercalcemia. The researchers suggested that the increase in basal levels and PTH secretion reserve in normal glands in the early postoperative period after pHPT surgery may be attributed to pre-synthesized and larger stored amounts of PTH (23).

Preoperative PTH levels and removed parathyroid glands’ volumes were significantly higher in patients without suppression at the 6th postoperative hour. Higher PTH levels in this group may be related to the higher volumes of removed adenomas. Previous studies have demonstrated a positive correlation between preoperative PTH level and removed adenoma volume (24, 25). In addition, some other studies have shown varying degrees of correlation between removed adenoma weight and preoperative PTH (26–29).

Risk factors for multigland disease have been researched and discussed for many years. Age is one of the most frequently discussed factors for multigland disease. In the study conducted by Wirowski et al., young age was found to be a risk factor for multiple gland disease (30). However, in the same study, when familial diseases are excluded, age is not a significant factor (30). In the study by Thier et al., it was shown that age did not make a significant difference in terms of single gland disease and multiple gland disease (31). In the study by Glen et al., although multi-gland disease was found to be significantly higher in patients over 60 years of age, it was not determined as an independent risk factor in multivariance analysis (32). In literature, age is one of the debatable risk factors of multigland disease. According to our results, mean age was significantly higher in group 2. Although the higher mean age in non-suppressed group shows us that persistence and, indirectly, multi-gland disease may be higher in older ages, we believe that more comprehensive studies are needed on this subject.

Although there is no consensus in current guidelines regarding the frequency of postoperative visits in patients with pHPT, it has been stated that this is a multifactorial issue, influenced by both the surgeon’s preference and patient-specific factors (1). Laurie et al. reported that a PTH level of <3 pmol/L (28.3 ng/L) at 4–6 hours on postoperative day 0 could predict surgical cure at 6 months with a sensitivity of 81.3% and a specificity of 100% (PPV: 100%, NPV: 20%, accuracy: 82.1%, AUC: 0.947). They suggested that this value could be used to identify patients who are likely to be cured after parathyroidectomy for pHPT at an early stage, thereby eliminating the need for continued biochemical monitoring (33).

In present study, a postoperative 6th-hour PTH level of <18 ng/L was significantly associated with surgical cure at 6 months (sensitivity: 61.9%, specificity: 73.7%, diagnostic accuracy: 66.7%; AUC: 0.68, p = 0.006). Given the markedly increased risk of persistent disease in patients without adequate PTH suppression following focused surgery or unilateral neck exploration, this parameter may be considered when determining the frequency of postoperative follow-up. In contrast, Laurie et al. reported that a PTH level <28.3 ng/L at 4–6 hours on postoperative day 0 predicted cure with an accuracy of 82.1%, sensitivity of 81.3%, specificity of 100%, PPV of 100%, and NPV of 20% (AUC: 0.947) (33). These differing results highlight the variability in diagnostic performance across studies and support the need for flexible postoperative monitoring strategies that account for clinical and biochemical diversity in pHPT patients.

The main limitation of present study is its retrospective design, which may affect the generalizability of the findings. Moreover, postoperative blood sampling was performed at a single time point, which may have restricted a more comprehensive evaluation of PTH kinetics throughout the postoperative period. Although functional testing, such as a calcium suppression test, was not included in the current study design, its use might be considered in future research to provide further physiological insight, especially in patients with borderline or unexpectedly low PTH levels. Further studies are still needed to differentiate between persistent pHPT patients with and without early PTH suppression. To the best of our knowledge, this is the first study to evaluate the association between early postoperative PTH suppression and persistent disease in pHPT patients who underwent focused or unilateral neck exploration. Patients who underwent bilateral neck exploration were excluded to eliminate the potential confounding effect of postoperative hypoparathyroidism, which could have influenced the outcomes.

## Conclusion

In conclusion, early postoperative PTH suppression is highly associated with surgical cure. The rate of pPHPT is significantly higher in non-suppressed patients. Therefore, in follow-up strategies of postoperative patients, the possibility of a remnant pathological gland should be considered especially in those without early PTH suppression.

## Data Availability

The original contributions presented in the study are included in the article/Supplementary Material. Further inquiries can be directed to the corresponding author.

## References

[1] Wilhelm SM Wang TS Ruan DT Lee JA Asa SL Duh QY. The American Association of Endocrine Surgeons guidelines for definitive management of primary hyperparathyroidism. JAMA Surg. (2016) 151:959–68. doi: 10.1001/jamasurg.2016.2310, PMID: 27532368

[2] Uludağ M Ünlü MT Köstek M Caliskan O Aygun N Isgor A. Persistent and recurrent primary hyperparathyroidism: etiological factors and pre-operative evaluation. Med Bull Sisli Etfal Hosp. (2023) 57:1–17. doi: 10.14744/SEMB.2023.39260, PMID: 37064844 PMC10098391

[3] Auguste L Attie J Schnaap D. Initial failure of surgical exploration in patients with primary hyperparathyroidism. Am J Surg. (1990) 160:333. doi: 10.1016/S0002-9610(05)80536-7, PMID: 2221229

[4] Yeh MW Wiseman JE Chu SD Duh QY Clark OH. Population-level predictors of persistent hyperparathyroidism. Surgery. (2011) 150:1113–9. doi: 10.1016/j.surg.2011.09.025, PMID: 22136829

[5] Peacock M. Calcium metabolism in health and disease. Clin J Am Soc Nephrol. (2010) 5:S23–30. doi: 10.2215/CJN.05910809, PMID: 20089499

[6] Bergenfelz A Ahrén B. Suppressibility of serum levels of PTH by calcium in the immediate postoperative period after surgery for primary hyperparathyroidism. World J Surg. (1993) 17:806–10. doi: 10.1007/bf01659104, PMID: 8109123

[7] Akerström G Rastad J Ljunghall S Norlén O. Cellular physiology and pathophysiology of the parathyroid glands. World J Surg. (1991) 15:672–80. doi: 10.1007/BF01665299, PMID: 1767532

[8] Bergenfelz A Valdermarsson S Ahrén B. Suppression by calcium of serum levels of intact parathyroid hormone in patients with primary hyperparathyroidism. Horm Res. (1993) 39:146–51. doi: 10.1159/000182715, PMID: 8262476

[9] Tohme JF Bilezikian JP Clemens TL Silverberg SJ Shane E. Suppression of parathyroid hormone secretion with oral calcium in normal subjects and patients with primary hyperparathyroidism. J Clin Endocrinol Metab. (1990) 70:951–6. doi: 10.1210/jcem-70-4-951, PMID: 2318950

[10] Nussbaum SR Thompson AR Hutcheson KA Gaz RD Wang CA. Intraoperative measurement of parathyroid hormone in the surgical management of hyperparathyroidism. Surgery. (1988) 104:1121–6., PMID: 3194839

[11] Medas F Cappellacci F Canu GL Calò PG. The role of Rapid Intraoperative Parathyroid Hormone (ioPTH) assay in determining outcome of parathyroidectomy in primary hyperparathyroidism: A systematic review and meta-analysis. Int J Surg. (2021) 92:106042. doi: 10.1016/j.ijsu.2021.106042, PMID: 34339883

[12] Yavuz S Simonds WF Weinstein LS Chen CC Reynolds JC Libutti SK. Sleeping parathyroid tumor: rapid hyperfunction after removal of the dominant tumor. J Clin Endocrinol Metab. (2012) 97:1834–41. doi: 10.1210/jc.2011-3030, PMID: 22508712 PMC3387414

[13] Barczyński M Konturek A Hubalewska-Dydejczyk A Cichon S Nowak W. Evaluation of Halle, Miami, Rome, and Vienna intraoperative iPTH assay criteria in guiding minimally invasive parathyroidectomy. Langenbecks Arch Surg. (2009) 394:843–9. doi: 10.1007/s00423-009-0510-z, PMID: 19529957

[14] Yeh R Tay YD Dercle L Corrias G Kulkarni K Kunstman JW. A simple formula to estimate parathyroid weight on 4D-CT, predict pathologic weight, and diagnose parathyroid adenoma in patients with primary hyperparathyroidism. AJNR Am J Neuroradiol. (2020) 41:1690–7. doi: 10.3174/ajnr.A6687, PMID: 32816774 PMC7583096

[15] Muzurović E Tomšić KZ Vujošević S Kravarusic J Vujović S Popović V. Parathyroid hormone and calcitonin response during the calcium infusion test in patients with primary hyperparathyroidism. Hormones (Athens). (2022) 21:261–70. doi: 10.1007/s42000-022-00353-2, PMID: 35102498

[16] Titon I Cailleux-Bounacer A Basuyau JP Do Cao C Lefebvre H Wémeau JL. Evaluation of a standardized short-time calcium suppression test in healthy subjects: interest for the diagnosis of primary hyperparathyroidism. Eur J Endocrinol. (2007) 157:351–7. doi: 10.1530/EJE-07-0132, PMID: 17766719

[17] Brown EM Wilson RE Thatcher JG Lechene CP Aurbach GD. Abnormal calcium-regulated PTH release in normal parathyroid tissue from patients with adenoma. Am J Med. (1981) 71:565–70. doi: 10.1016/0002-9343(81)90207-2, PMID: 7282744

[18] Zettinig G Kurtaran A Prager G Niederle B Dudczak R. ‘Suppressed’ double adenoma–a rare pitfall in minimally invasive parathyroidectomy. Horm Res. (2002) 57:57–60. doi: 10.1159/000057949, PMID: 12006722

[19] Siperstein A Berber E Barbosa GF Mack E Gill I Dutta S. Predicting the success of limited exploration for primary hyperparathyroidism using ultrasound, sestamibi, and intraoperative parathyroid hormone analysis of 1158 cases. Ann Surg. (2008) 248:420–8. doi: 10.1097/SLA.0b013e3181859f71, PMID: 18791362

[20] Heath H 3rd Sizemore CW Carney JA. Preoperative diagnosis of occult parathyroid hyperplasia by calcium infusion in patients with multiple endocrine neoplasia, type 2a. J Clin Endocrinol Metab. (1976) 43:428–35. doi: 10.1210/jcem-43-2-428, PMID: 950371

[21] Mayer GP Hurst JG. Sigmoidal relationship between parathyroid hormone secretion rate and plasma calcium concentration in calves. Endocrinology. (1978) 102:1036–42. doi: 10.1210/endo-102-4-1036, PMID: 744006

[22] Marx SJ Lasker RD Brown EM Aurbach GD Spiegel AM McMillin JM. Secretory dysfunction in parathyroid categories from a neonate with severe primary hyperparathyroidism. J Clin Endocrinol Metab. (1986) 62:445–9. doi: 10.1210/jcem-62-2-445, PMID: 3941166

[23] Bergenfelz A Ahrén B. Intraoperative secretion of intact parathyroid hormone and amino-terminal parathyroid hormone fragments from normal parathyroid glands associated with solitary parathyroid adenoma. World J Surg. (1997) 21:30–4. doi: 10.1007/s002689900189, PMID: 8943174

[24] Filser B Uslar V Weyhe D Kersting S. Predictors of adenoma size and location in primary hyperparathyroidism. Langenbecks Arch Surg. (2021) 406:1607–14. doi: 10.1007/s00423-021-02179-9, PMID: 33928428 PMC8370949

[25] Bindlish V Freeman JL Witterick IJ Asa SL Gullane PJ. Correlation of biochemical parameters with single parathyroid adenoma weight and volume. Head Neck. (2002) 24:1000–3. doi: 10.1002/hed.10165, PMID: 12410535

[26] Papadakis M Weyerbrock N Zirngibl H Schlosser K. Correlation of perioperative biochemical variables with single adenoma weight in patients with primary hyperparathyroidism. BMC Surg. (2020) 20:303. doi: 10.1186/s12893-020-00922-5, PMID: 33256695 PMC7708903

[27] Stern S Mizrachi A Strenov Y Knaanie A Benbassat C Shpitzer T. Parathyroid adenoma: a comprehensive biochemical and histological correlative study. Clin Otolaryngol. (2017) 42:381–6. doi: 10.1111/coa.12761, PMID: 27696726

[28] Kandil E Alabbas H Tufaro AP Maloney P Moulthrop TH Slakey DP. The impact of baseline intact parathyroid hormone levels on severity of primary hyperparathyroidism and outcomes in patients undergoing surgery. Arch Otolaryngol Head Neck Surg. (2010) 136:147–50. doi: 10.1001/archoto.2009.225, PMID: 20157060

[29] Mózes G Curlee KJ Rowland CM Olson JA Jr Farley DR Grant CS. The predictive value of laboratory findings in patients with primary hyperparathyroidism. J Am Coll Surg. (2002) 194:126–30. doi: 10.1016/S1072-7515(01)01139-5, PMID: 11848628

[30] Wirowski D Lammers BJ Pohl P Schott M Goretzki PE. Does multiple gland disease in primary hyperparathyroidism correlate with age or sex? Langenbecks Arch Surg. (2009) 394:885–90. doi: 10.1007/s00423-009-0521-9, PMID: 19533167

[31] Thier M Daudi S Bergenfelz A Almquist M Nordenström E Hellman P. Predictors of multiglandular disease in primary hyperparathyroidism. Langenbecks Arch Surg. (2018) 403:103–9. doi: 10.1007/s00423-017-1647-9, PMID: 29294178 PMC5805794

[32] Glenn JA Yen TW Javorsky BR Wilson SD Evans DB Wang TS. Association between body mass index and multigland primary hyperparathyroidism. J Surg Res. (2016) 202:132–8. doi: 10.1016/j.jss.2015.12.055, PMID: 27083959

[33] Laurie BD Leong D Nguyen H Dodd J O’Neill CJ. Day zero parathyroid hormone levels predict cure after parathyroidectomy. ANZ J Surg. (2025). doi: 10.1111/ans.19411, PMID: 39887490

